# Adult-onset Still's disease after ChAdOx1 nCoV-19 vaccine: a possible association

**DOI:** 10.4322/acr.2021.403

**Published:** 2022-11-07

**Authors:** Laíssa Fiorotti Albertino, Isac Ribeiro Moulaz, Tammer Ferreira Zogheib, Martina Zanotti Carneiro Valentim, Ketty Lysie Libardi Lira Machado

**Affiliations:** 1 Universidade Federal do Espírito Santo (UFES), Medical School, Vitória, ES, Brasil; 2 Hospital Evangélico de Vila Velha, Department of Internal Medicine, Vila Velha, ES, Brasil; 3 Vitoria Apart Hospital, Department of Infectology, Vitoria, ES, Brasil; 4 Universidade Federal do Espírito Santo (UFES), Department of Rheumatology, Internal Medicine, Vitória, ES, Brasil

**Keywords:** Adenovirus Vaccines, COVID-19 Vaccines, Still's Disease, Adult-Onset

## Abstract

With emergent Sars-Cov-2, a highly transmissive virus that caused millions of deaths worldwide, the development of vaccines became urgent to combat COVID-19. Although rare, important adverse effects had been described in a hypothetical scenario of immune system overstimulation or overreaction. Still’s disease is a rare inflammatory syndrome of unknown etiology. It manifests as a cytokine storm, mainly IL-18 and IL-1β, and presents itself with fever spikes, joint pain, maculopapular evanescent salmon-pink skin rash, and sore throat, among other symptoms. Here, we report a case of a 44-year-old healthy male who developed adult-onset Still’s disease (AOSD) with atypical symptoms after both doses of ChAdOx1 nCoV-19 vaccine with 3 months of dose interval. The medical team suspected Still's disease and started prednisone 1 mg/kg (40mg). The next day the patient showed a marked improvement in articular and chest pains and had no other fever episodes. Therefore, he was discharged to continue the treatment in outpatient care. On the six-month follow-up, the patient was free of complaints, and the progressive corticoid withdrawal plan was already finished.

## INTRODUCTION

With emergent Sars-Cov-2, a highly transmissive virus that caused millions of deaths worldwide, the development of vaccines became urgent to combat COVID-19. Since the vaccine race has slowed after several viable vaccine options, the focus of vaccine research has shifted toward minimizing potential side effects. Although rare, important adverse effects have been described in hypothetical scenarios of immune system overactivation, which may trigger the onset of autoimmune disease.^1^ In this setting, adult-onset Still’s disease (AOSD) was reported only a few times after the BNT162b2 mRNA COVID-19 (Pfizer),^2^ mRNA-1273 COVID-19 vaccine (Moderna),^3^ and ChAdOx1 nCoV-19 (AstraZeneca) vaccines.^4^

AOSD is a rare inflammatory syndrome of unknown etiology that manifests as a cytokine storm, mainly IL-18 and IL-1β. It presents itself with fever spikes, joint pain, maculopapular evanescent salmon-pink skin rash, and sore throat, among other signs and symptoms.^5^ The hyperinflammatory state, the role of IL-1, and response to corticosteroid therapy and IL-1 inhibition are some similarities observed in inflammatory storm pathogenesis in COVID-19 and AOSD.

## CASE REPORT

We report the case of a 44-year-old previously healthy male who developed AOSD after receiving the first dose of the ChAdOx1 nCoV-19 vaccine. He received the anti-COVID-19 AstraZeneca vaccine (an adenoviral vector vaccine) at the end of July 2021. On the day following vaccination, the patient developed a fever of 38°C and chills lasting 48 hours. He also developed odynophagia, myalgia, arthralgia, and right cervical lymphadenopathy five days later.

As a result of the increasing severity of his symptoms, he presented to the emergency department (ED) at a private hospital. The patient reported pain near the right scapula and diarrhea, in addition to the symptoms previously described, and a fever-like (not measured) episode the day before the consultation. Following the physical examination, the patient was afebrile, with oropharyngeal hyperemia (without exudates), and tenderness near the right scapula. Antigen testing for COVID-19 was negative, and a computed tomography (CT) scan showed no abnormalities. The patient was discharged with symptomatic treatment.

Five days later (11 days after vaccination), the patient returned to the ED complaining of left hypochondrium pleuritic pain that irradiated to left hemithorax, hyporexia, class III dyspnea according to the New York Heart Association (NYHA) classification, and fever (>38°C). Following the physical examination, he was afebrile and displayed a salmon-pink rash in the infrasternal region and abdomen.

Abdominal CT scans revealed left pleural effusion and a small amount of free fluid in the pelvis. A 2D echocardiogram showed a 75% left ventricular ejection fraction, mild mitral valve prolapse with mild regurgitation, and mild pericardial effusion. CT angiography of the chest revealed small bilateral pleural effusions, which were larger in the left pleural space, causing compressive atelectasis on the corresponding pulmonary segments. Polymerase chain reaction (PCR) and antigen testing for SARS-CoV-2 were performed to rule out acute COVID-19. The patient was admitted to the internal medicine division for further investigation. Serum hematologic evaluation displayed leukocytosis of 21,400/ml (reference value, RV, of 4000-11000/ml) with 83% neutrophils, a normocytic and normochromic anemia (hemoglobin of 11.4 g/dL), C-Reactive protein (CRP) of 362.2 mg/L (RV of <1 mg/L) and serum ferritin levels of 1650 ng/mL (RV 16-243 ng/mL).

The following day, the patient still had evidence of an evanescent salmon-pink maculopapular rash in the infrasternal region and left flank, a palpable cervical lymph node, pain in the right proximal interphalangeal joints (without arthritis), a positive squeeze test on the left foot, and lower limb edema 2+/4+. Venous Doppler ultrasonography ruled out bilateral deep vein thrombosis.

Due to the worsening of his symptoms, such as persistent fever of 38°C, leukocytosis, and cutaneous lesions (due to the patient's own manipulation), empirical antibiotics (vancomycin and piperacillin-tazobactam) were initiated, which were withdrawn after 6 days since the patient showed no sign of improvement or infection (blood and urine cultures were negative). The patient was tested for toxoplasmosis, hepatitis B and C viruses, human immunodeficiency virus (HIV), syphilis, cytomegalovirus (CMV), and Epstein-Barr virus, which were negative. Pleural effusion liquid analysis showed elevated levels of adenosine deaminase (60 U/L), the protein level of 4,71 g/dL, neutrophils of 91%, lymphocytes of 5%, and returned negative results for fungal and bacterial agents. The pleural liquid was exudative accordingly to Light's criteria [Pleural lactate dehydrogenase (770 U/L)/Serum lactate dehydrogenase ratio (240 U/L) ≥ 0.6]. During hospitalization, the patient was treated with analgesics (codeine and tramadol), antipyretics, diuretics, and anti-inflammatory drugs.

During the last 15 days of hospitalization, imaging examinations were repeated and showed no alteration. Since the patient improved, no fever was reported during the previous 48h, no dyspnea or hyporexia, and decreased pain; he received hospital discharge with naproxen (500 mg 12/12h). He was oriented to continue his treatment in outpatient care for further investigation without progress.

The patient received the second dose of the same ChAdOx1 nCoV-19 vaccine three months after the first dose. After 18 days, he was hospitalized due to an unintentional loss of 13 kg in 2 months (with no change in eating habits), an intermittent fever of 38ºC, cervical posterior bilateral lymphadenopathy, pruritus rash, asthenia, and continued arthralgia on the shoulders, hands, and knees. Shoulder ultrasonography ruled out the presence of arthritis.

Upon physical examination, he had a heart rate of 110 beats per minute, a palpable, painless, and movable lymph node in the right posterior cervical area, an extensive salmon-pink rash on the back, and right wrist soreness and edema. Laboratory results showed an erythrocyte sedimentation rate of 100 mm/h (reference range of 0-15 mm in first hour), platelet count of 744,000 p/mm3, leukocytes of 16,700/ml with 77% neutrophils, CRP 204.1 mg/L, hemoglobin of 9.1 g/dL, ferritin of 645.7 ng/mL. Antinuclear antibodies were absent, and the rheumatoid factor was negative.

The Rheumatology team raised the hypothesis of adult-onset Still's disease and initiated oral prednisone at a dose of 1 mg/kg/day (40 mg). The next day, the patient showed marked improvement in articular and chest pain and had no other febrile episodes. Therefore, the patient was discharged to continue treatment in outpatient care. Upon the one-month follow-up, the patient regained 5 kg and was free of complaints; hence, progressive corticosteroid withdrawal was conducted, and the patient remained asymptomatic after a six-month follow-up.

## DISCUSSION

Here, we report a rare case of AOSD after vaccination with the COVID-19 adenoviral vector vaccine. The patient reported symptoms such as fever, arthralgia, spiking fever, skin rash, throat ache, odynophagia, myalgia, lymphadenopathy, pericarditis, pleuritis, and presented elevated ferritin levels, erythrocyte sedimentation rate, and C-reactive protein levels, which are all in accordance with the Yamaguchi (sensitivity of 96.2% 80.6% specificity 92.1%) and Fautrel criteria (sensitivity 80.6% and specificity 98.5%)^6^ adding to an extensive mandatory work-up to rule out infections and other inflammatory conditions that mimic AOSD.

Although post-anti-COVID-19 vaccine AOSD was the presumed diagnosis, we cannot exclude the possibility that the timing concerning vaccination was coincidental. The onset of the symptoms 1 day after the first dose and the recurrence of symptoms reinforce the causal relationship between the AOSD and anti-COVID-19 vaccination. Also, the repetition of symptoms 18 days after the second dose supports the idea that this represented a new onset of AOSD rather than a hyperinflammatory response to vaccination.

The exact triggers of damage-associated molecular patterns (DAMPs) and pathogen-associated molecular patterns (PAMPs) that underlie the autoimmune response in AOSD are controversial and not yet known. Therefore, the process of eliminating similar medical conditions has to be carefully conducted over a considerable amount of time. After the immune activation, overproduction of IL-1β (as well as IL-6, IL-8, IL-17, IL-18, and TNF-α) by caspases, excessive levels of ferritin stimulated by macrophages, enhancement of neutrophil extracellular traps (NET), dysfunctional natural killer cells, high levels of T-helper Th1, Th17, and alarmins, promote a proinflammatory environment that favors the abnormal autoimmune response.^6^

Cases of AOSD after COVID-19 infection are rare but present in the literature.^7^ The SARS-CoV-2 infection triggers a storm of cytokines and production of autoantibodies,^8^ leading to similarities (to some extent) in the immune response resulting from both disease conditions, wherein IL-1, IL-6, and IL-18 play a key role.^1^ In this scenario, it has been suggested that exposure to COVID-19 vaccine antigens can produce a less intense, similar immune response sufficient to trigger the antigen-induced autoimmunity by unclear mechanisms.^9^^,^^10^ Few cases of Still’s disease-onset after Influenza^11^ and Pneumococcal^12^ vaccination have also been reported. These correlations highlight the possibility of autoimmune activation after infection or vaccination. However, the triggering factors and mechanisms for AOSD development require further investigation.

Viral infection and vaccine antigen exposure are thought to induce or trigger an autoimmune reaction by molecular mimicry or bystander activation.^11^^,^^13^ Molecular mimicry can lead to autoimmunity when partial homology exists between the viral antigen and self-antigen, acting as a mistaken trigger for immune activation; also, a cytokine storm due to macrophage activation syndrome (MAS) can destroy both normal and infected cells, possibly resulting in the release of sequestrated antigens. This process activates preexisting autoreactive T cells, which further enhance cytokine production, resulting in the progression of the immune reaction.^11^^,^^14^ Once molecular mimicry is thought insufficient to induce autoimmune diseases, the bystander mechanism is probably involved.^15^ Adding to that, our patient had a symptomatic event 1 day after the first dose of the vaccine, suggesting the involvement of a mechanism other than molecular mimicry since this mistaken immune activation requires weeks to induce an autoimmune reaction.

Finally, corticosteroids are the gold standard treatment of AOSD. High prednisone doses (≥40 mg, 0.8 mg/kg) are associated with effective disease control and quicker remission than lower dosages.^16^ Also, the use of other medications as immunosuppressants should be considered in managing the disease. In this case, the patient showed complete remission of symptoms with corticosteroids alone.

## CONCLUSION

Despite being rare, adult-onset Still’s disease can be triggered by the adenoviral vector ChAdOx1 nCoV-19 vaccine as an acute and persistent side effect that must be recognized early in the treatment and prevention of potential complications. Even though there is a low risk of developing AOSD after vaccination, the authors reiterate that vaccination is the most effective way to reduce the severity of COVID-19. Further studies are required to establish autoimmune cross-activation mechanisms after COVID-19 vaccine administration.
